# Stoichiometric Representation of Gene–Protein–Reaction Associations Leverages Constraint-Based Analysis from Reaction to Gene-Level Phenotype Prediction

**DOI:** 10.1371/journal.pcbi.1005140

**Published:** 2016-10-06

**Authors:** Daniel Machado, Markus J. Herrgård, Isabel Rocha

**Affiliations:** 1 Centre of Biological Engineering, University of Minho, Braga, Portugal; 2 The Novo Nordisk Foundation Center for Biosustainability, Technical University of Denmark, Horsølm, Denmark; EMBL-Heidelberg, GERMANY

## Abstract

Genome-scale metabolic reconstructions are currently available for hundreds of organisms. Constraint-based modeling enables the analysis of the phenotypic landscape of these organisms, predicting the response to genetic and environmental perturbations. However, since constraint-based models can only describe the metabolic phenotype at the reaction level, understanding the mechanistic link between genotype and phenotype is still hampered by the complexity of gene-protein-reaction associations. We implement a model transformation that enables constraint-based methods to be applied at the gene level by explicitly accounting for the individual fluxes of enzymes (and subunits) encoded by each gene. We show how this can be applied to different kinds of constraint-based analysis: flux distribution prediction, gene essentiality analysis, random flux sampling, elementary mode analysis, transcriptomics data integration, and rational strain design. In each case we demonstrate how this approach can lead to improved phenotype predictions and a deeper understanding of the genotype-to-phenotype link. In particular, we show that a large fraction of reaction-based designs obtained by current strain design methods are not actually feasible, and show how our approach allows using the same methods to obtain feasible gene-based designs. We also show, by extensive comparison with experimental 13C-flux data, how simple reformulations of different simulation methods with gene-wise objective functions result in improved prediction accuracy. The model transformation proposed in this work enables existing constraint-based methods to be used at the gene level without modification. This automatically leverages phenotype analysis from reaction to gene level, improving the biological insight that can be obtained from genome-scale models.

## Author Summary

Genome-scale models of metabolism enable the exploration of the phenotypic landscape of an organism. Unlike probabilistic approaches such as genome-wide association studies, these models describe the mechanistic link between genotype and phenotype, predicting the response to genetic and environmental perturbations. However, this connection is hampered by the complexity of gene-protein-reaction associations. In this work, we implement a model transformation method that untangles this complexity by allowing gene-wise phenotype predictions using genome-scale models. The transformed model explicitly accounts for the individual flux carried by the enzyme or subunit encoded by each gene. Previously published simulation methods are automatically leveraged by this transformation, enabling new features such as the formulation of objectives and constraints at the gene/protein level. We demonstrate the application of different kinds of analysis and simulation methods, showing in each case how the gene-wise formulation can result in higher prediction accuracy in comparison to experimental data and improve the biological insight that can be obtained from available models.

## Introduction

The advances in high-throughput sequencing techniques and genome annotation methods have enabled the construction of genome-scale models for hundreds of organisms [1]. At the same time, the constraint-based framework, with its wide variety of methods, has become a widely used tool to perform *in silico* experiments and predict cellular responses to different kinds of genetic and environmental perturbations [2, 3]. Studies using constraint-based models cover a wide range of applications from biomedical research to industrial biotechnology, including the study of cancer metabolism [4], drug target discovery for cancer cell lines [5] and pathogenic microorganisms [6], and the design of microbial cell factories [7] and synthetic microbial communities [8].

Understanding the complex relation between the genotype and phenotype of an organism is a fundamental part of systems biology research. Unlike statistical approaches such as genome-wide association studies (GWAS) [9], genome-scale reconstructions provide a mechanistic link between genotype and phenotype. The first component of this link is a list of gene-protein-reaction (GPR) associations that determines the set of metabolic reactions encoded in the genome. Another component is the stoichiometric matrix representing these reactions. This matrix is at the core of every constraint-based method, allowing the computation of the metabolic phenotype as described by metabolic fluxes at steady-state. Navigating back and forth in the space of genotype-to-phenotype relationships is hampered by the complex association between genes, enzymes and reactions. From the perspective of the central dogma of biology the simplest genetic mechanism is: one gene—one protein—one function. However, most GPR associations in a genome-scale metabolic network are quite complex due to the presence of enzyme complexes (multiple genes—one protein), isozymes (multiple proteins—one function) and promiscuous enzymes (one protein—multiple functions).

Since most constraint-based methods do not explicitly account for GPR associations, they can only provide analysis at the reaction level. For instance, simulating a steady-state flux distribution predicts the rates of all metabolic reactions for a given phenotypic state, but fails to elucidate the contribution of individual genes/enzymes to that phenotype. GPR associations, typically implemented as Boolean rules, can be used to interpret the results of constraint-based analysis in an *ad-hoc* fashion. This is the case in rational strain design, where optimization procedures are used to find optimal interventions to maximize the production of a given compound [7, 10]. With a few exceptions [11–13], such methods can only compute reaction-based modifications that must be translated to gene-level modifications *a posteriori*, without guarantee that the optimality of the predicted phenotype is preserved. Undesired side-effects may arise if any of the target reactions involve promiscuous enzymes.

In this work, we present a model transformation that generates a stoichiometric representation of GPR associations that can be directly integrated into the stoichiometric matrix. We show that the results obtained with the transformed model are consistent with those obtained from reaction-level models, and highlight the advantages of performing different kinds of analysis at the gene level. We also propose new variants of existing methods that take advantage of this representation to formulate gene-wise objective functions and test their predictive ability using experimental datasets.

## Results

**Fig 1:**
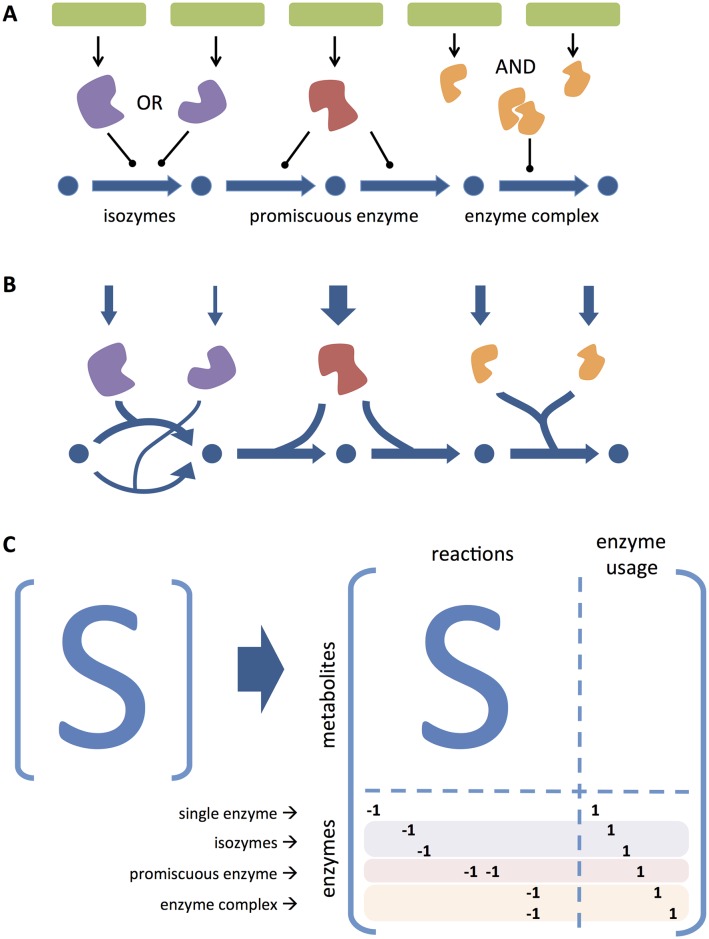
GPR transformation method. Transformation of GPR associations to a stoichiometric representation: a) Boolean representation of different types of GPR associations including isozymes (purple), promiscuous enzymes (red), protein complexes (orange); b) Continuous representation accounting for the individual flux carried by each enzyme (the arrow thickness represents a possible solution for the distribution of fluxes); c) Extended stoichiometric matrix highlighting the occurrence of particular GPR association cases highlighted in panel a.

The proposed model transformation to encode GPR associations into the stoichiometric matrix is depicted in Fig 1. This transformation changes the Boolean representation of gene states (on/off) to a real-valued representation. Essentially, the enzyme (or enzyme subunit) encoded by each gene becomes a species in the model, and the participation of an enzyme in a reaction is encoded by adding the respective (pseudo-)species to the left-hand side of that reaction (Fig 1b). Reversible reactions and reactions catalyzed by multiple isozymes are decomposed into individual reactions. A set of artificial reactions, denoted as *enzyme usage* reactions (*u*), are added to the model. For each gene, this variable accounts for the total amount of flux carried by the respective enzyme (or enzyme subunit). This model transformation can be represented by an extended stoichiometric matrix (Fig 1c).

**Fig 2:**
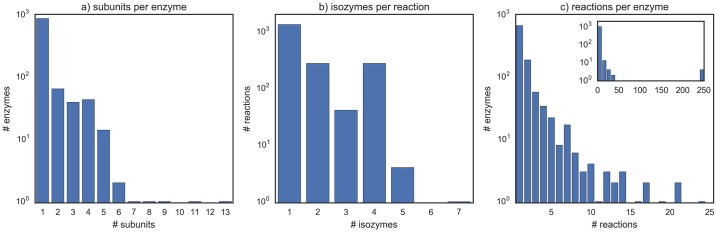
Complexity of GPR associations. Frequency distribution of the GPR associations in the iAF1260 model with respect to: a) number of subunits per enzyme complex; b) number of isozymes per reaction; c) number of reactions catalyzed per enzyme.

The iAF1260 genome-scale model for *E. coli* was used as case study [14]. A statistical analysis of the structure of the GPR associations reveals the complexity of the underlying topology (Fig 2). Over 16% of the enzymes are formed by protein complexes (up to 13 subunits), with many subunits being common to different complexes. About one third of the reactions (31%) are catalyzed by multiple isozymes (up to 7), and more than two thirds (72%) are catalyzed by at least one promiscuous enzyme. Four genes (*phoE, ompC, ompN, ompF*) stand out as outliers with regard to promiscuity due to their participation in nearly 250 transport reactions.

The proposed transformation was applied to the model after pre-processing to remove blocked reactions (determined by flux variability analysis for aerobic growth on glucose minimal medium). The simplified model contains 914 genes, 1532 reactions, and 1032 metabolites (including external metabolites and respective exchange reactions). After transformation, the extended stoichiometric matrix contains 3853 (pseudo-)reactions and 1946 (pseudo-)metabolites. The increase in the size of the stoichiometric matrix is caused by introducing the genes as species in the model, decomposing reversible and isozyme-catalyzed reactions, and introducing the artificial “enzyme usage” reactions (see Methods). In the following subsections, we exemplify the application of this extended model to different types of constraint-based analysis.

### Flux distribution prediction

#### Wild-type

Simulating steady-state flux distributions using flux balance analysis (FBA) is the most common application of constraint-based models. FBA requires the definition of an objective function based on evolutionary assumptions [15]. The maximization of rate of biomass formation has shown agreement with experimental observations [16] and is often used. The solution obtained by FBA simulation for the transformed model is the same as for the original model, since the transformed model does not contain additional constrains compared to the original model. However, the solution vector obtained from the FBA simulation is now more informative due to the *enzyme usage* variables, which determine the amount of flux carried by each individual enzyme.

**Fig 3:**
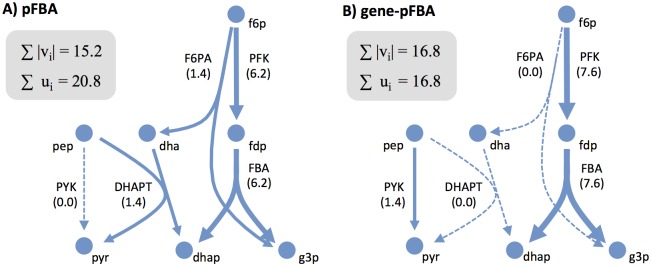
pFBA simulation of a single solution. Difference in simulation results for: A) pFBA and B) gene-pFBA. Simulations performed using the iAF1260 model with default model conditions (aerobic growth on glucose minimal medium with a maximum glucose uptake rate of 10 mmol/gDW/h). The values of the respective objective functions for this particular subsystem are indicated.

FBA results are usually affected by solution degeneracy, as different flux distributions can have the same objective value. Parsimonious FBA (pFBA) is a two-step variant of FBA that determines the flux distribution that satisfies the optimal objective while also minimizing total absolute flux through all reactions. It is based on the assumption that the cell tries to achieve its goal with the most efficient allocation of resources [17]. Although pFBA is usually implemented as the minimization of reaction fluxes, with our extended representation it can be more naturally formulated as the minimization of enzyme usage (see methods). Testing both methods for a simple simulation (with default model conditions) resulted in similar solutions, except for different choices in a few central carbon reactions (Fig 3). It can be observed that pFBA also uses an alternative route to convert fructose-6-phospate (f6p) to dihydroxyacetone-phosphate (dhap) and glyceraldehyde-3-phosphate (g3p), which has the side-effect of converting phosphoenolpyruvate (pep) to pyruvate (pyr), eliminating the need to use pyruvate kinase (PYK). This removes one step in the functional pathway, decreasing the total sum of fluxes. The gene-based approach does not use this alternative route, resulting in a slightly higher sum of fluxes, but reducing the total enzyme allocation. The increased enzyme usage in the first case is caused by the DHAPT reaction, which is catalyzed by a complex formed by 5 subunits, therefore increasing the overall enzyme allocation for that pathway. Considering that glycolytic fluxes are commonly measured *in vivo*, if such a considerable deviation of flux would occur, it would have been frequently reported. Hence the pFBA prediction seems less plausible (a systematic comparison with experimental data will be considered later in this section).

We compared our simulation results with those obtained using the recent *E. coli* ME-model [18] under the same conditions (S4 Fig). ME-models account for the operational costs of the translational machinery required to produce metabolic enzymes In these models, the reaction fluxes are coupled to the synthesis rate of the respective enzymes. We observe a significant correlation between the translation rates predicted by the ME-model and the enzyme usage predicted with our gene-pFBA approach (Pearson R = 0.84, P<5e-57). Interestingly, the flux distribution predicted with the ME-model seems to be degenerate with regard to the alternative routes detected between pFBA and gene-pFBA, with small parameter perturbations leading to one or the other. This indicates that the cost of the two alternatives must be similar. In fact, although our method assumes that the PYK route is cheaper, this reaction is catalyzed by two isozymes (PykA, PykF), which are both tetramers. Since the number of subunit copies per enzyme is not reported in GPR associations, the enzyme usage is underestimated for multimeric enzymes. If this information becomes systematically available in genome-scale reconstructions, it can be easily accounted for in the stoichiometric coefficients of the extended matrix.

#### Deletion mutants

Multiple alternative methods to standard FBA simulations have been proposed for prediction of phenotypic effects of gene deletions in the constraint-based framework [19–21]. Such methods assume that the mutant cell will minimize its regulatory and metabolic adjustment with respect to the wild-type phenotype. Again, these assumptions can be represented in a more biologically meaningful way at the gene/enzyme level using the transformed model. Hence, we implemented gene-based versions of MOMA and linearMOMA (see methods).

**Fig 4:**
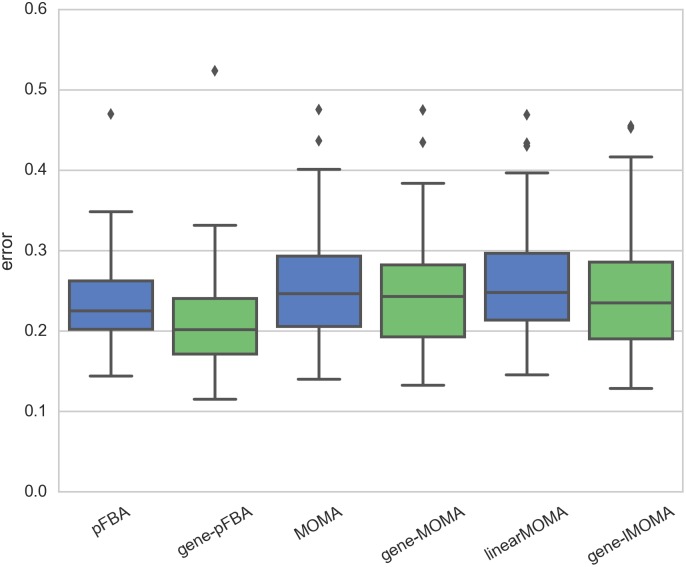
Knockout simulation benchmark. Distribution of the normalized prediction error for the flux distribution of 24 single deletion mutants (Ishii2007 dataset) generated by pFBA, MOMA, linearMOMA (blue) and their respective gene-level counterparts (green).

In order to evaluate the simulation accuracy of the proposed methods we performed a systematic evaluation using a fluxomics dataset for 24 single gene *E. coli* mutants [22] (see methods). Fig 4 shows a comparison of the prediction error for each method across all mutant strains. It can be observed that, in general, all reaction-based methods have higher prediction error than their gene-level counterparts. In particular, gene-pFBA stands out as the most accurate method in this case study.

#### Over/under-expression

The effects of modulating gene or protein expression can also be simulated by constraint-based methods. This is performed by imposing lower/upper bounds in the flux of the respective reactions. However, the limitations of this approach must be carefully considered. It assumes a linear effect between the expression level of a gene and the rate of the respective reactions (i.e. fully transcriptional control), which is only the case under particular circumstances [23]. Also, it does not consider the potential side-effects of enzyme promiscuity. The gene-level formulation alleviates these problems, since the constraints can be imposed directly on the *enzyme usage* variable of the respective gene, rather than directly at the reaction level (see methods).

**Fig 5:**
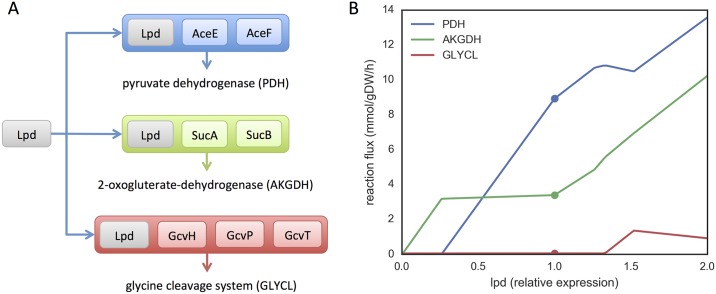
Effects of over/under-expression. Modulating the expression of the *ldp* gene: a) reaction participation of the enzyme subunit encoded by *lpd*; b) effect of modulating the expression of *lpd* in the rate of the respective reactions (the circles represent the reference state for the wild-type simulation).

To illustrate this concept, we simulated the over/under-expression of the *lpd* gene (Fig 5, see methods). This gene encodes an enzyme subunit that participates in three different reactions. It is possible to observe that the effect of gene modulation on the respective reactions is not linear. For instance, the gradual down-regulation of *lpd* is predicted to initially decrease the rate of pyruvate dehydrogenase (PDH) and only afterwards affect the rate of 2-oxoglutarate-dehydrogenase (AKGDH). Using reaction-based constraints to simulate the over/under-expression of any of these reactions would predict a linear effect between the regulation level and the respective flux, disregarding the potential redistribution of flux between promiscuous enzymes and isozymes. Considering the large fraction of promiscuous enzymes (Fig 2c), this non-linear redistribution of flux becomes particularly relevant for strain design methods that account for over/under-expression at the reaction level [24–29]. This particular application will be further discussed in the strain design section.

### Gene essentiality analysis

Gene essentiality analysis consists of the identification of conditionally lethal gene deletions [30]. This type of analysis can be used to find drug targets for pathogenic microbes [31] and particular types of cancer cells [5]. It can also be used to improve model reconstructions by comparison with experimental data, and to exclude undesirable gene deletions from the search space of strain design algorithms.

Gene essentiality analysis is usually performed by simulating the knockout of each gene in two stages, which requires evaluating the respective GPRs followed by FBA simulation to test the model for growth. With the extended stoichiometric matrix, gene essentiality analysis can be directly performed by flux variability analysis (FVA) (see methods). For each gene, the flux range of the respective *enzyme usage* variables indicates the minimum and maximum amount of flux that can be carried in the given experimental conditions (S1 Fig). In this case, any gene with a minimal enzyme usage above zero for a given minimal biomass production is essential. This approach can also be generalized to find synthetic lethal pairs by systematic computation of the minimum sum of fluxes of all pairs of *enzyme usage* variables.

This analysis is more informative then traditional determination of essentiality (binary test) as it reveals the minimal (and maximal) flux that can be carried by each enzyme. With this approach one can also determine “blocked” genes (i.e. genes encoding enzymes that cannot be used under any conditions), which can be used to guide the model reconstruction process. Furthermore, one can use the shadow price and reduced costs information to analyse the sensitivity of the results with respect to internal (biological) and external (environmental) constraints. For instance, the non-zero shadow prices for an essential gene represent the set of precursor metabolites that cause the essentiality, whereas the reduced costs of exchange reactions represent the effect of changing the medium composition with regard to essentiality.

### Flux space sampling

Random sampling of the flux solution space is a suitable strategy to analyse all possible physiological states described by a model [32]. Like FVA, it is an unbiased method to describe the flux solution space. However, while FVA only describes the admissible flux range for a given reaction, random flux sampling generates a probability distribution for each reaction, providing insight into the shape of the solution space. Flux sampling has been used to analyse global properties of metabolic networks [33] and to determine flux variation in perturbed conditions [34].

One limitation of flux sampling is that it does not account for the flux load distribution between isozymes or the overall flux carried by promiscuous enzymes. With the extended representation, it is now possible to analyse flux sampling results at the gene/enzyme level. An illustrative example for a model of core metabolism of *E. coli* [35] is presented (S2 Fig). Flux sampling results are compared for two conditions: a wild-type phenotype and a succinate producing phenotype (see methods). It is possible to observe significant differences between both phenotypes at the gene level. There is an increased flux in enzymes involved in lower glycolysis and the glyoxylate shunt, and an overall decrease of flux for enzymes in the pentose-phosphate pathway and the respiratory chain. Note that one can observe gene level differences that would not be captured by purely reaction-based sampling, such as the different utilization of *Lpd* relative to other enzyme subunits (*AceE, AceF, SucA, SucB*) given its simultaneous participation in different enzyme complexes.

Flux sampling at the gene level can be used to guide rational strain design, since non-overlapping sampling distributions for a given gene between wild-type and the desired mutant indicate that the flux carried by the respective enzyme must necessarily change. We compared these results with those obtained by strain design methods that account for modulation of gene expression [25–28]. Some of the most significant changes observed (deletion of sdh* and overexpression of frd*, *ppc*, and *aceA*) are commonly proposed interventions to increase succinate production. It is also possible to observe some extent of agreement between our sampling results and gene expression measurements of succinate producing mutants [36], most notably the down-regulation of *aceE, aceF, icd, pykA* and *pykF*.

### *Omics* data integration

The continuous improvement of high-throughput techniques to measure different kinds of *omics* data has fostered the development of constraint-based methods that make use of these data to improve predictions. In a recent work, we evaluated several methods for integration of transcriptomics (and proteomics) data into constraint-based simulations, and observed that none of the methods resulted in consistent improvement of flux predictions compared to simple FBA simulation under the assumption of optimal growth and parsimonious enzyme usage [37]. This limitation arises from the underlying assumption of proportionality between gene expression and reaction rates, which does not seem to be generally valid [38, 39].

It seems natural to reformulate some of these methods to take advantage of the flux simulation at the enzyme level. In this work, we propose gene-wise reformulations of two commonly used methods, GIMME and E-Flux [40, 41]. In the reformulated versions, the expression level of a gene is mapped to its respective *enzyme usage* variable (see methods). The original and reformulated versions of the methods were evaluated using two experimental datasets containing transcriptomics and fluxomics data [22, 42] (see methods).

**Fig 6:**
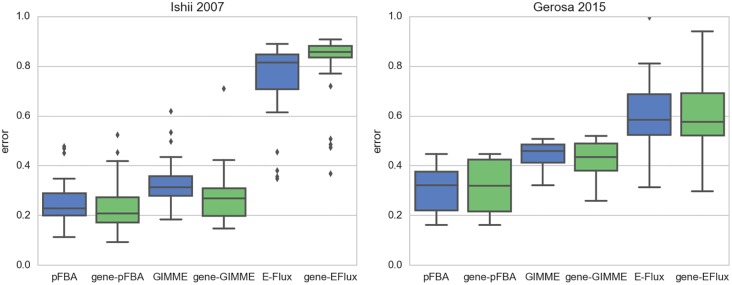
Transcriptomics-based simulation benchmark. Distribution of the normalized prediction error for intracellular fluxes generated by pFBA, GIMME, E-Flux (blue) and their respective gene-level counterparts (green), by integration of gene expression data from two datasets (Ishii2007, Gerosa2015).

Similarly to our previous study, the results reveal that none of the transcriptomics-based methods outperforms pFBA (Fig 6). However, as observed earlier, gene-pFBA shows better performance than pFBA for the Ishii dataset. The gene-wise version of GIMME is generally more accurate than the original version in both datasets. This improvement can be attributed to the fact that the gene-wise formulation is less affected by the lack of correlation between gene expression and reaction rates. No improvement could be observed for the gene-wise version of E-Flux.

### Elementary mode analysis

Elementary mode analysis provides an unbiased description of the flux solution space of a metabolic network by determining all minimal pathways that can operate at steady-state, so-called elementary flux modes (EFMs) [43]. Elementary mode analysis reveals multiple properties of metabolic networks, including pathway yields, reaction usage frequency, and correlated reaction sets [44]. Common applications include analysis of cellular robustness [45], detection of fragility points in metabolic networks as potential drug targets [46], and elimination of undesired phenotypes to design optimal cell factories [47].

**Fig 7:**
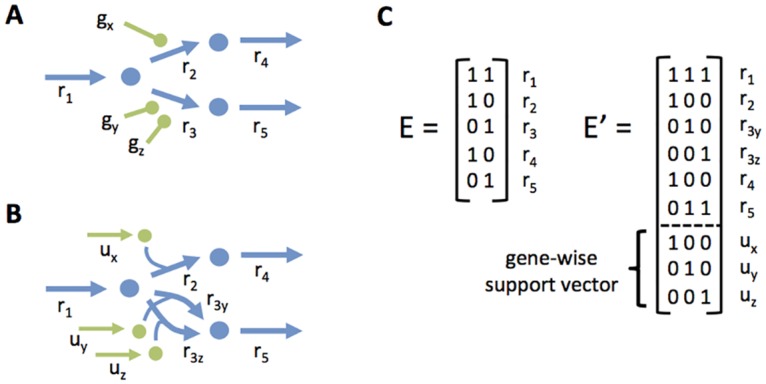
EFM analysis overview. Gene-wise elementary mode analysis illustrated: a) Toy model of a small metabolic network, where *r*_2_ is catalyzed by an enzyme encoded by gene *g*_*x*_ and *r*_3_ is catalyzed by two isozymes encoded by genes *g*_*y*_ and *g*_*z*_ b) Transformed version of the toy model; c) Elementary modes calculated for the original network (*E*), and the transformed network (*E*′).

Since EFM computation does not account for GPR associations, they do not entirely reflect the topology of a metabolic network, disregarding that a promiscuous enzyme is a common link between different pathways and that isozymes provide alternative routes within the same pathway. Our stoichiometric representation of GPRs solves this problem by explicitly accounting for this complexity in the computation of EFMs. This concept is illustrated in Fig 7. Although EFM computation algorithms differ with regard to specific implementation details, the manipulation of support vectors is a common denominator. Support vectors are binary representations of the minimal set of reactions included in an EFM. With the network transformation, the artificial *enzyme usage* reactions become part of the support vector of EFMs, being automatically computed by any EFM computation algorithm. This extended support vector contains a gene-wise representation of each EFM, denoting the genes that participate in the given pathway.

**Fig 8:**
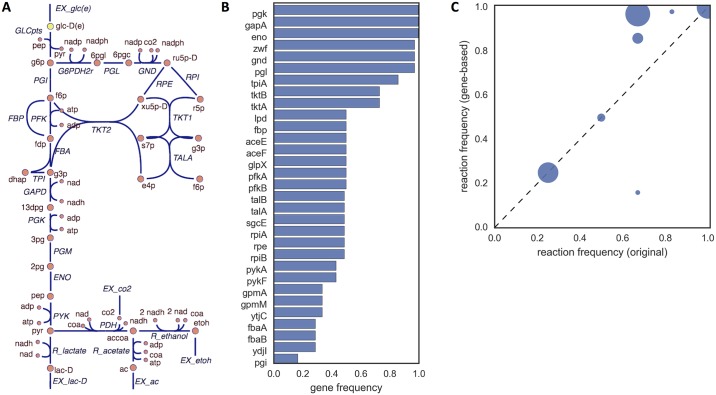
Gene-wise EFM analysis results. Gene-wise elementary mode analysis results: a) Simplified central carbon model of *E. coli* including glycolysis, pentose-phosphate pathway, and the production of lactate, acetate and ethanol; b) frequency analysis of the pathway participation of each gene; c) comparison of the reaction frequency between the original and the transformed model (circle size indicates the number of reactions in that region), elements above or below the diagonal indicate, respectively, an increase or decrease in the frequency of those reactions when GPR associations are considered.

We applied this analysis to a simplified central carbon model of *E. coli* (Fig 8a). The model contains a total of 12 EFMs. After transformation the number of EFMs raises to 11085. This drastic increase is caused by splitting isozymes into separate reactions, which leads to a large combination of possible routes. Fig 8b shows the gene participation in the set of EFMs. It can be observed that *pgk*, *gapA*, and *eno* participate in every pathway. These would be the best targets in a drug design application. On the other hand, *pgi* has the lowest participation (16.4%). The deletion of this gene would cause the least impact in the network. We also compared the frequency of each reaction in the original model and the transformed model (Fig 8c). There is an overall increase in the frequency of reactions in the pentose-phosphate pathway due to the alternative routes created by the presence of isozymes. The frequency of glycolytic reactions remains the same, with the exception of PGI with a significant decrease (from 67% to 16%). These results show that accounting for GPRs can shed a different perspective on the relative importance of different reactions, with a potential impact in methods that search for the most important pathway disruptions to block undesired phenotypes [46, 47]. Although the increase in the number of EFMs hampers large-scale EFM computation, this approach is still amenable to the application of EFM-based methods that do not require complete enumeration of the full EFM set (see discussion).

### Rational strain design

Designing optimal cell factories for production of industrially relevant compounds is one of the most common applications of constraint-based modeling. Genome-scale models can be used to guide rational strain design by predicting the phenotype of mutant strains, which can be iteratively improved until economically viable product yields, titers and productivities are attained. The countless combinations of manipulations that could be tested require the implementation of powerful optimization methods to search the genetic design space [7, 10]. Although a large number of methods (∼50) have been published so far, very few allow gene-based modifications [11–13]. The vast majority of methods determine optimal sets of reaction-based modifications (deletions or up/down-regulations) that must be *a posteriori* translated into gene-based designs for *in vivo* implementation. Given that enzyme promiscuity can affect a major fraction of the reactions in a model (Fig 2), it can be expected that many reaction-based designs will result in undesired side-effects when implemented at the gene level.

**Fig 9:**
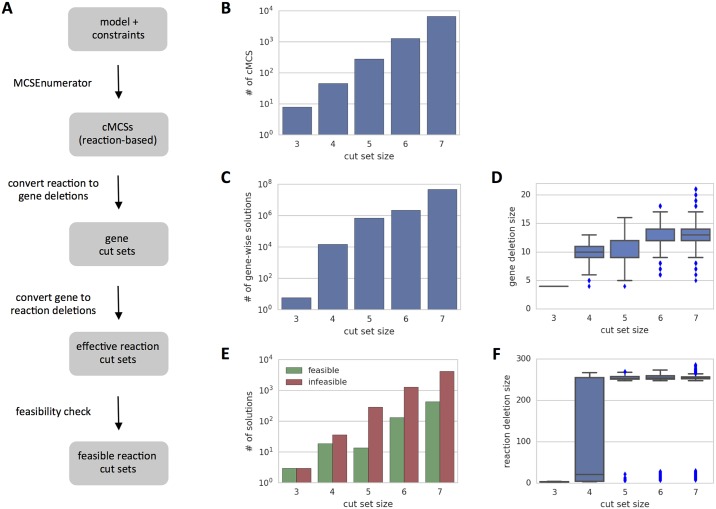
Reaction-based strain design with MCSEnumerator. Reaction-based strain design using MCSEnumerator for growth-coupled ethanol production in *E. coli* under anaerobic conditions (*μ* ≥ 0.001 *h*^−1^, *Y*_*eth*/*glc*_ ≥ 1.4). a) Required workflow to generate and validate reaction-based cMCSs; b) Computed cMCSs up to size 7; c) Total number of potential gene-wise solutions for the computed cMCSs; d) Average number of gene deletions for each cut sizes; e) Total number of feasible and infeasible solutions for each cut size; f) Average number of effective reaction deletions for each cut size.

MCSEnumerator is a recently published method that enumerates all minimal sets of reaction deletions up to a given size, so-called constrained minimal cut sets (cMCSs), that are guaranteed to couple product formation to growth [48]. This method represents a breakthrough in the field, allowing unprecedented enumeration of the design space at the genome scale. We reproduced the results presented by the authors for growth-coupled ethanol production (scenario 1), and performed a deeper analysis of the feasibility of the strain designs when mapping the reaction-based solutions to gene-based ones (Fig 9).

Given that any gene encoding a subunit of an enzyme complex can be deleted to disable the respective function, the number of potential designs significantly increases when converting reaction to gene deletions (Fig 9c). This mainly results from the presence of reactions catalyzed by multiple complex isozymes. For instance, formate hydrogen lyase (FHL) can be catalyzed by two different complexes, with 11 and 7 subunits each, resulting in 77 possible combinations of gene deletions to disable this reaction. Other notable cases include the PTS system (57 combinations) and ATP synthase (44 combinations). It can also be observed that the total number of required gene deletions can be significantly larger than the respective number of reaction deletions. For instance, a strain design of 4 reaction deletions may require up to 13 gene deletions (Fig 9d).

In order to test the feasibility of each design, accounting for possible side-effects, we calculated the actual set of reactions effectively disabled by the gene deletions required to implement a given cMCS. We then evaluated each phenotype and observed that only a small fraction of the original set of solutions (∼7%) are valid with respect to the original production constraints (Fig 9e). This drastic effect is mainly caused by the deletion of highly promiscuous enzymes (such as those involved in transporters), which can result in the deletion of hundreds of side-effect reactions (Fig 9f).

**Fig 10:**
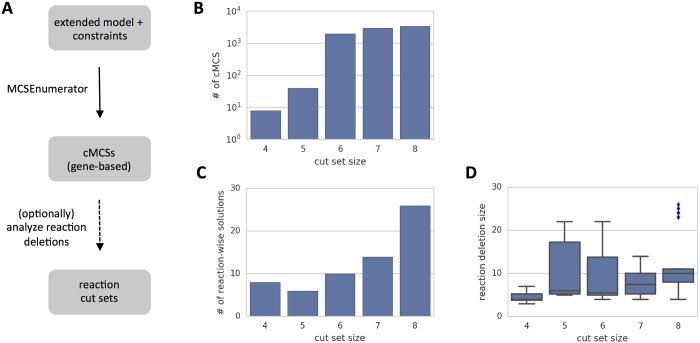
Gene-based strain design with MCSEnumerator. Gene-based strain design using MCSEnumerator for growth-coupled ethanol production in *E. coli* under anaerobic conditions (*μ* ≥ 0.001 *h*^−1^, *Y*_*eth*/*glc*_ ≥ 1.4): a) Steps required to generate and analyse gene-based cMCSs; b) Computed cMCSs up to size 8; c) Total number of reaction-based solutions generated by the gene-wise cMCSs; d) Average number of reaction deletions for each cut size.

The shortcomings of reaction-based design can be avoided by directly searching for gene-based designs. We applied MCSEnumerator to the transformed model and computed all minimal gene-based cut sets up to 8 deletions (Fig 10). It can be observed that the total number of gene-based designs is now much lower. In this case, all designs are necessarily feasible since all potential side-effects are implicitly accounted for. Nonetheless, we confirmed the feasibility of each design by testing the respective reaction deletions in the original model. Note that the total number of reaction-based designs is actually lower, since different gene cut sets generate the same reaction deletions (Fig 10c). Furthermore, it can be observed that the number of deleted reactions is generally higher than the number of respective gene deletions without compromising the feasibility of the strain design (Fig 10d).

Finally, we tested cRegMCSs, a recent extension of MCSEnumerator that accounts for reaction up/down-regulation [29]. With our approach it is possible to apply constraints directly at the gene level, correctly accounting for the limitations discussed earlier and without any modification to the original method. Using a core metabolic model for *E. coli* and the same production goals as before, thousands of designs were found with as few as 3 gene manipulations (see S3 Fig).

## Discussion

We presented a model transformation technique that integrates GPR associations into the stoichiometric matrix of a metabolic model. This allows the application of a wide-range of constraint-based methods to the transformed model, automatically extending these methods from reaction to gene-based analysis. This was illustrated by application of different kinds of methods to a genome-scale model of *E. coli*. We illustrated how gene essentiality can be determined by flux variability analysis, and how flux sampling can be used to reveal the flux solution space at the enzyme level, taking into account the flux load distribution between isozymes and the cumulative flux of promiscuous enzymes.

We also proposed reformulated versions of existing methods for phenotype prediction (gene-pFBA, gene-MOMA) and methods for integration of transcriptomics data (gene-GIMME, gene-EFlux), where the objective function and model constraints are reformulated in a gene-based representation. Systematic evaluation of these methods by comparison with experimental flux data revealed that the new methods have higher average prediction accuracy than their original versions. This shows that not only it is possible to reformulate the evolutionary assumptions that support these methods in a more biologically meaningful way, but doing so can actually improve flux predictions.

Predicting the effect of gene-level modifications with our extended model representation can be directly performed by constraining the respective *enzyme usage* variables. This representation is more accurate than applying these constraints at reaction level. For instance, deleting a single isozyme has no effect in a reaction-based representation, whereas the gene-based representation accounts for the redistribution of flux among other isozymes of the affected reaction. We also demonstrated the non-trivial consequences of performing over/under-expression of promiscuous enzymes. Forcing an increased or decreased activity in a promiscuous enzyme results in an uneven redistribution of flux among the respective reactions in order to reach a new optimal phenotype. This effect would not be predictable by current methods.

The integration of GPR associations reveals a distinct topology of the metabolic network. It accounts for the fact that promiscuous enzymes provide a common link between different pathways, and that isozymes provide alternative routes within a pathway. We demonstrated how this affects the characterization of the network in terms of its elementary flux modes. The EFMs computed for the transformed model have an extended support vector that accounts for the genes that must be active to support the respective pathways. This enables different types of elementary mode analysis (e.g: frequency distribution, minimal cut sets) to be performed at the gene level. One disadvantage of the extended representation is the combinatorial explosion of EFMs caused by the decomposition of isozyme-catalyzed reactions. Nonetheless, gene-wise EFM computation is still amenable to the application of EFM-based methods that do not require complete enumeration of the full EFM set [49–51].

Rational strain design is likely the application where our proposed transformation will be most useful. The large number of strain design methods published so far (∼50) can be automatically used to search for gene-based manipulations without any modification to the underlying method by first applying our transformation to the model. This provides several significant advantages compared with reaction-based design. First, reaction-based design ignores the side-effects of enzyme promiscuity and requires careful *post hoc* analysis of proposed designs. We used MCSEnumerator to determine all minimal reaction cut sets (up to size 7) for ethanol production in *E. coli*. We tested the feasibility of these cut sets when GPR associations are considered, and observed that only less than 10% of the solutions are actually feasible. Since many of these reaction-based solutions would also be determined by other strain design methods, this problem affects such methods as well. Another advantage of computing gene-based designs is to effectively minimize the number of genetic interventions, which is the desired scenario for *in vivo* construction of the mutant strain. We observed that determining minimal reaction deletions can result in a large number of gene deletions. On the other hand, gene-based design effectively minimizes the number of gene deletions regardless of the number of affected reactions. In terms of computation costs, one would expect the increased model size to be detrimental for efficiency. However, the complexity of MILP problems (used in MCSEnumerator and many other strain design methods) is related to the number of decision variables (deletion targets). Since the number of genes is lower than the number of reactions, we were able to compute gene-based cMCS up to size 8 with the same computational resources used to compute reaction-based cMCSs up to size 7.

It is important to note that other strategies to integrate GPR associations in constraint-based simulations have been proposed. All methods that integrate transcriptional regulatory networks and metabolic networks need to account for GPRs, as they provide the connection layer between the two types of network. In SR-FBA, genes are encoded as integer variables and the GPR associations are encoded as linear inequalities, transforming the model into an MILP formulation [52]. This approach was also implemented in the strain design method OptORF [12], and later generalized by the TIGER toolbox that automates the generation of integrated models [53]. This approach differs from ours by representing genes as integer variables and implementing the GPRs as MILP constraints, which limits the applicability of the formulation and increases computational demands significantly. A recent method called Logic Transformation of Model (LTM) implements a network transformation that expands a metabolic network in such a way that the gene-reaction association matrix (GRAM) unambiguously represents GPR associations [54]. The GRAM is binary matrix often used to encode GPR associations. It loses information by ambiguously representing AND and OR relationships. The artificial reactions introduced by LTM solve this ambiguity, and the GRAM can be used to map gene deletion vectors directly to reaction deletion vectors. The authors show how this method can also be used to leverage strain design methods from reaction to gene level, and present two such methods (OptGeneKnock and FastGeneSL). However, LTM presents some disadvantages compared to our approach. The artificial metabolites and reactions introduced are mathematical artifacts without biological meaning. The gene levels are interpreted in a binary fashion and can only be used to compute reaction knockouts. This reduces the applicability of the approach to deletion-based methods. Also, it requires a reformulation of each method to account for the transformed GRAM. Our transformation generates a purely metabolic representation of GPR-encoded models that can be represented using standard formats (such as SBML) and directly used by the myriad of metabolic modeling tools currently available [55].

Finally, it is important to note the potential pitfalls of using a gene-based analysis. In this work, we used a highly curated genome-scale model of *E. coli* [14]. Nowadays, automatic reconstruction tools can generate new models in a short time [56–58]. While different methods exist to curate the model at the reaction level (with regard to elemental balance, thermodynamic feasibility, gap filling, *etc*), GPR associations can only rely on the quality of gene annotations. With our approach, incorrect assignment of GPR associations will be reflected in the extended stoichiometric matrix and may generate misleading results. On the positive side, our proposed approach may also facilitate the development of new methods for curation of GPRs (e.g.: gene-wise gap-filling). Another potential disadvantage of performing gene-level analysis is the increased computational cost of working with an extended stoichiometric matrix. Although the effect is barely noticeable for single simulations (∼ 1.5-fold increase in simulation time) it may be undesirable for more computationally intensive types of analysis such as random flux sampling or flux variability analysis.

### Final remarks

The integration of GPRs directly into the stoichiometric matrix enables bidirectional analysis between the gene and reaction levels. In one direction it is possible to observe the impact of gene perturbations on reaction fluxes. In the other direction one can perturb the environmental conditions and observe the required adaptations at the gene level. The complexity of GPR associations and their evolution has been recently analysed considering the role of environmental adaptation in driving enzyme specificity [59]. A recent reconstruction of the *underground* metabolism of *E. coli* revealed an even larger number of metabolic reactions available in the cell as a result of enzymatic side activities, playing an important role in the fitness landscape of the organism [60].

Our framework provides a mechanistic link between genotype and phenotype and should facilitate the development of new methods to integrate multi-omics datasets into genome-scale models, as well as methods to integrate metabolism with other biological processes. In this work, we explored the reformulation of previously published simulation methods with gene-wise constraints. It would be interesting to explore other suitable applications, such as the formulation of simulation methods that account for enzyme production costs [61, 62].

A new generation of genome-scale models and simulation methods is on the rise [63]. This includes genome-scale models that account for gene expression and protein production [18, 64, 65], models that account for protein structure [66], and methods that predict the effect of genetic variation in protein function [67]. While such detailed models are not readily available for every organism, our method provides a suitable approach to leverage existing models to a new level.

All the source code, models, and generated data are publicly available in the following repository: https://github.com/cdanielmachado/GPRTransform.

## Methods

### Models and tools

Unless otherwise stated, all simulations were performed using the iAF1260 genome-scale metabolic model for *E. coli* [14] and the Gurobi solver (version 6.5). The core metabolism version of this model [35] was used for random flux sampling and strain design with cRegMCS [29].

### Model transformation

The model transformation to explicitly include GPRs in the stoichiometric matrix (as exemplified in Fig 1) is defined as follows. Let *S* be the original stoichiometric matrix, *v* the steady-state flux vector (after decomposition of reversible reactions and isozyme-catalyzed reactions), and *ub* the respective vector of flux bounds, such that *S* ⋅ *v* = 0 and 0 ≤ *v* ≤ *ub*. The extended stoichiometric matrix *S*′, flux vector *v*′, and flux bounds *ub*′ are defined as:

$$S^{\prime }=\left|\begin{array}{c} \begin{array}{ll} S & 0\\ S_{\text{gpr}} & I_{k} \end{array} \end{array}\right|\;v^{\prime }=\left|\begin{array}{c} v\\ u \end{array}\right|\;ub^{\prime }=\left|\begin{array}{c} ub\\ +\infty \end{array}\right|$$

where *S*_gpr_ is the stoichiometric representation of GPRs (with *s*_*i*, *j*_ = −1 if gene *i* participates in reaction *j*), *I*_*k*_ is the identity matrix for *k* genes, and *u* is the enzyme usage vector. This transformed model can be readily used by any kind of constraint-based method with the general form:

$$\begin{array}{l} \text{min}/\text{max }f\left(v^{\prime }\right)\\ \;s.t.\\ \;S^{\prime }\cdot v^{\prime }=0\\ \;0\leq v^{\prime }\leq ub^{\prime } \end{array}$$

where *f* is a given objective function.

### Gene-wise method reformulation

The gene-wise reformulations differ from the original methods by expressing the objective functions and genetic constraints using the enzyme usage variables instead of reaction fluxes, and are defined as follows.

#### gene-pFBA

Let $v_{\text{growth}}^{\text{max}}$ be the maximum growth rate determined by FBA:

$$\begin{array}{l} \text{min}\sum_{i=1}^{k}u_{i}\\ \;s.t.\\ \;S^{\prime }\cdot v^{\prime }=0\\ \;0\leq v^{\prime }\leq ub^{\prime }\\ \;v_{\text{growth}}=v_{\text{growth}}^{\text{max}} \end{array}$$

#### gene-MOMA

Let *D* be the set of deleted genes, and *u*^wt^ the wild-type enzyme usage vector obtained by pFBA:

$$\begin{array}{l} \text{min}\sum_{i=1}^{k}{\left(u_{i}-u_{i}^{\text{wt}}\right)}^{2}\\ \;s.t.\\ \;S^{\prime }\cdot v^{\prime }=0\\ \;0\leq v^{\prime }\leq ub^{\prime }\\ \;u_{i}=0\;\forall i\in D. \end{array}$$

#### gene-lMOMA

Let *D* be the set of deleted genes, and *u*^wt^ the wild-type enzyme usage vector obtained by pFBA:

$$\begin{array}{l} \text{min}\sum_{i=1}^{k}\left|u_{i}-u_{i}^{wt}\right|\\ \;s.t.\\ \;S^{\prime }\cdot v^{\prime }=0\\ \;0\leq v^{\prime }\leq ub^{\prime }\\ \;u_{i}=0\;\forall i\in D \end{array}$$

#### Over/under-expression

Let *λ* be the relative expression level of gene *i* and *u*^wt^ the wild-type enzyme usage vector obtained by pFBA:

$$\begin{array}{l} \text{max }v_{\text{growth}}\\ \;s.t.\\ \;S^{\prime }\cdot v^{\prime }=0\\ \;0\leq v^{\prime }\leq ub^{\prime }\\ \;u_{i}\geq \lambda \cdot u_{i}^{\text{wt}}\text{ if }\lambda >1\\ \;u_{i}\leq \lambda \cdot u_{i}^{\text{wt}}\text{ if }\lambda <1 \end{array}$$

#### gene-GIMME

Let *e* be the gene expression vector, *e*_cutoff_ the 25th percentile of *e*, and $v_{\text{growth}}^{\text{max}}$ the maximum growth rate determined by FBA:

$$\begin{array}{l} \text{min}\sum_{i=1}^{k}\left(c_{i}\cdot u_{i}\right)\\ \;s.t.\\ \;S^{\prime }\cdot v^{\prime }=0\\ \;0\leq v^{\prime }\leq ub^{\prime }\\ \;v_{\text{growth}}=0.85\cdot v_{\text{growth}}^{\text{max}}\\ \;c_{i}=\{\begin{array}{ll} e_{\text{cutoff}}-e_{i} & \text{if }e_{\text{cutoff}}>e_{i}\\ 0 & \text{otherwise} \end{array} \end{array}$$

#### gene-EFlux

Let *e* be the gene expression vector and $v_{\text{glc}}^{\text{exp}}$ the experimentally determined glucose uptake rate:

$$\begin{array}{l} \text{(step 1)}\\ \;v\leftarrow \text{max }v_{\text{growth}}\\ \;s.t.\\ \;S^{\prime }\cdot v^{\prime }=0\\ \;0\leq v\leq ub\\ \;0\leq u_{i}\leq \frac{e_{i}}{\text{max }\left(e\right)}\forall i\\ \text{(step 2)}\\ \;v\leftarrow v\cdot \left(v_{\text{glc}}^{\text{exp}}/v_{\text{glc}}\right) \end{array}$$

### Benchmark

The methods to predict mutant phenotypes (pFBA [17], MOMA [19], linearMOMA, gene-pFBA, gene-MOMA, gene-lMOMA) were tested and compared with fluxomics data from the Ishii2007 dataset [22] that includes 24 single deletion mutants in chemostat cultivation at D = 0.2 *h*^−1^. In each case, the glucose uptake rate is constrained to the experimental value. The predicted fluxes are then compared to the experimental values, and the normalized prediction error is calculated as follows:

$$\text{error}=\frac{\left|\right|v^{\text{sim}}-v^{\text{exp}}\left|\right|}{\left|\right|v^{\text{exp}}\left|\right|}$$

where *v*^exp^ are the experimental fluxes, *v*^sim^ are the simulated fluxes for the experimentally measured reactions, and the vector norm is the *l*_1_-norm (Manhattan distance).

The methods for integration of gene expression data were tested using the transcriptomics and fluxomics data from two multi-omics datasets for *E. coli* [22, 42]. For the Ishii2007 dataset, all experimental conditions were used (wild-type at 5 different dilution rates and the 24 deletion mutants). The Gerosa2015 dataset includes data from shake flask cultivation under 8 different carbon sources. In this case, we constrained the uptake rate of the respective carbon source.

The prediction error for transcriptomics-based methods was also calculated as described above. However, we observed that for this kind of methods, the degeneracy of the optimal solution can influence the prediction error, hampering the reproducibility of results. To address this problem, in all the methods we add a second step that, after each simulation, determines the optimal solution with the smallest norm.

### Gene essentiality

Gene essentiality for each gene *i* was determined by flux variability analysis of the respective *enzyme usage* variable as follows:

$$\begin{array}{l} \text{min}/\text{max }u_{i}\\ \;s.t.\\ \;S^{\prime }\cdot v^{\prime }=0\\ \;0\leq v^{\prime }\leq ub^{\prime }\\ \;v_{\text{growth}}\geq 0.1\cdot v_{\text{growth}}^{\text{max}} \end{array}$$

where $v_{\text{growth}}^{\text{max}}$ is the maximal growth rate determined by FBA.

### Flux sampling

Random flux sampling was performed using the artificially centered hit-and-run (ACHR) method of the COBRA toolbox [68]. The wild-type phenotype was sampled under the assumption of a minimum biomass yield of 90% of the maximum theoretical value. The succinate producing mutant was sampled using a minimum of 10% for the biomass yield, and a minimum of 90% of the maximum succinate production (at 10% of biomass yield). Each phenotype was sampled for 10000 flux distributions with a step size of 100 jumps per sample.

### EFM computation

EFMs for the simplified glycolysis model were computed using CellNetAnalyzer version 2015.1 [69].

### MCSEnumerator

MCSEnumerator [48] was tested using the API interface from CellNetAnalyzer. We used the same model that was applied in the original publication (a version of iAF1260 customized for anaerobic growth). The list of targetable reactions was also defined as in the original publication. For the gene-wise version, the transformation was applied to the model, and the list of targetable reactions was defined to be the list of *enzyme usage* reactions. Computations were performed with CPLEX 12.6.3 using a 6-core Intel i7 processor with 64 GB of RAM.

### cRegMCS

cRegMCS [29] was tested using the API interface from CellNetAnalyzer. The problem setup was performed similarly to MCSEnumerator, except in this case the *E. coli* core model [35] was used due to the higher computational cost of the method. The number of regulatory steps was set to 3 levels for every gene (except those participating in futile cycles).

## Supporting Information

S1 Fig Gene essentiality analysis results. Gene essentiality determined by the flux variability analysis of the *enzyme usage* variables, calculated for a minimum biomass production of 10% of the maximum theoretical yield on glucose minimal media.
(TIFF)

S2 Fig Random flux sampling results. Flux sampling results for the core metabolism of *E. coli*. For each gene, the curves represent the probability distribution of the flux carried by the respective enzyme. Two scenarios are considered: wild-type phenotype near optimal growth (blue curves) and succinate overproduction near optimal yield (green curves). Genes where the blue and green distributions do not overlap are targets for modulation of gene expression.
(TIFF)

S3 Fig Gene-wise cRegMCS results. Gene-based strain design using cRegMCS for growth-coupled ethanol production in *E. coli* under anaerobic conditions (*μ* ≥ 0.001 *h*^−1^, *Y*_*eth*/*glc*_ ≥ 1.4): a) size of the solution pool for each cut size; b) total number of interventions of each type in the solution pool.
(TIFF)

S4 Fig ME-model simulation results. Comparison between the protein translation rates predicted by the ME-model and the respective enzyme usage predicted with gene-pFBA for a wild-type strain growing under aerobic conditions on glucose minimal medium with a glucose uptake rate of 10 mmol/gDW/h.
(TIFF)
