# Supplementary figures and images for: Stoichiometric Representation of Gene–Protein–Reaction Associations Leverages Constraint-Based Analysis from Reaction to Gene-Level Phenotype Prediction
Source: PLoS Comput Biol. 2016 Oct 6;12(10):e1005140. doi: 10.1371/journal.pcbi.1005140 (PMC5053500; doi:10.1371/journal.pcbi.1005140)

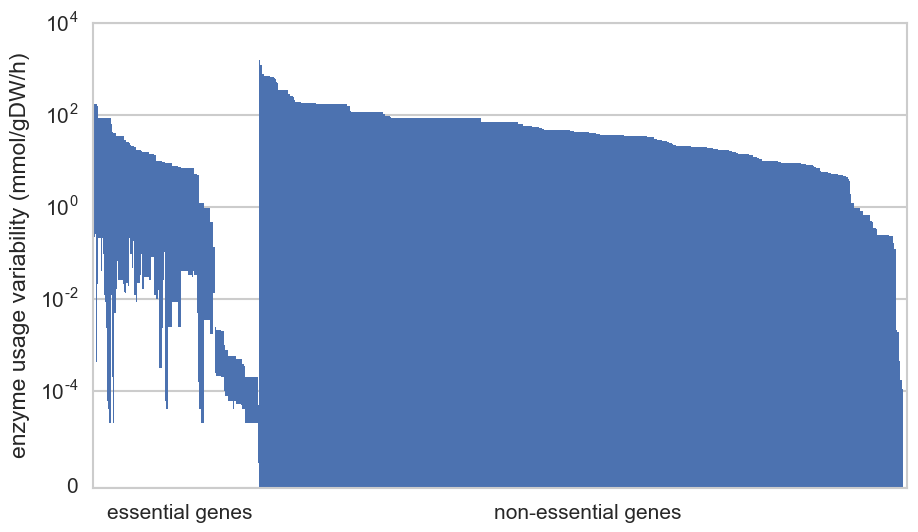

Supplement: S1 Fig — Gene essentiality determined by the flux variability analysis of the enzyme usage variables, calculated for a minimum biomass production of 10% of the maximum theoretical yield on glucose minimal media. (TIFF) [file pcbi.1005140.s001.tiff]

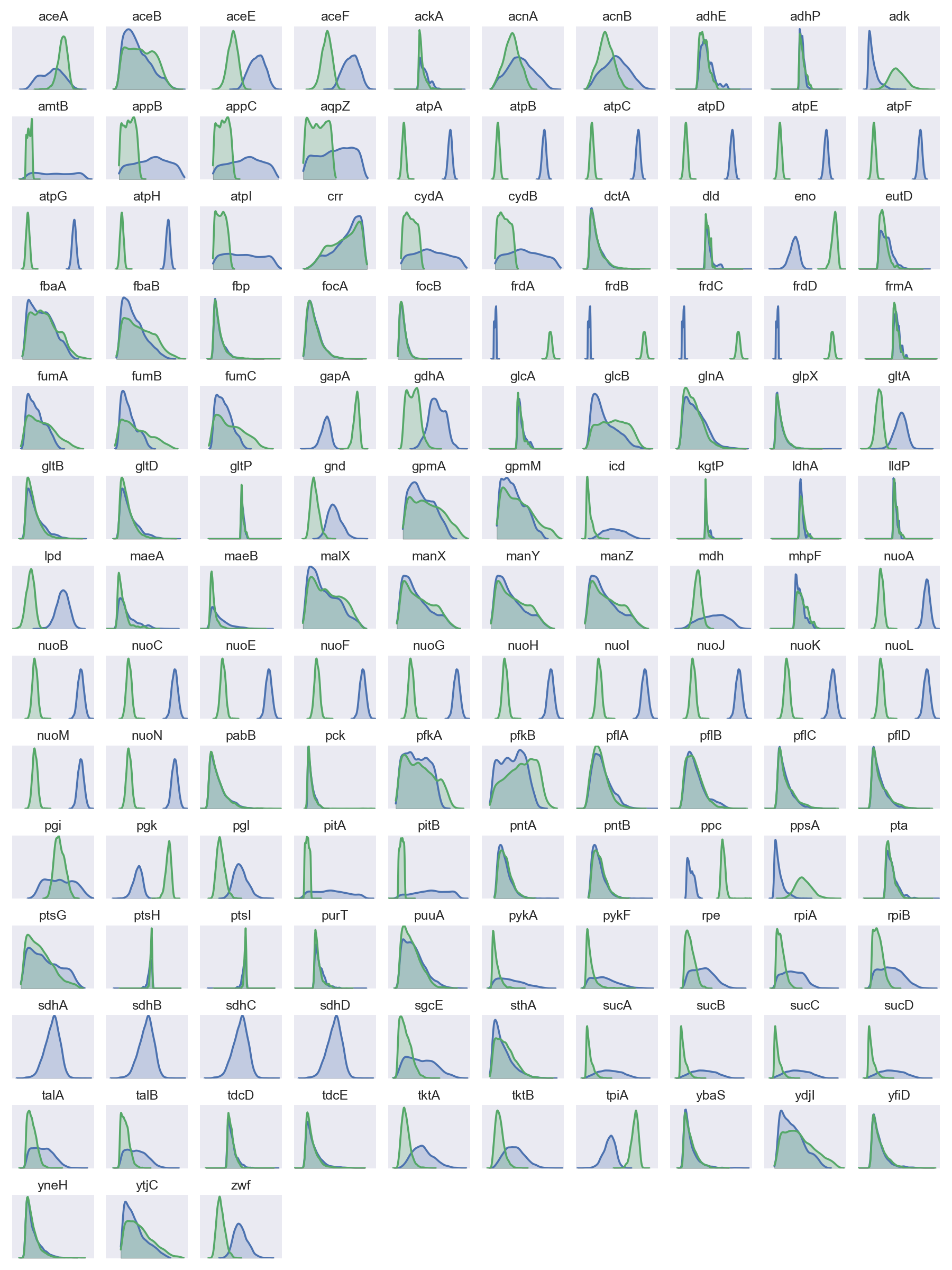

Supplement: S2 Fig — Flux sampling results for the core metabolism of E. coli. For each gene, the curves represent the probability distribution of the flux carried by the respective enzyme. Two scenarios are considered: wild-type phenotype near optimal growth (blue curves) and succinate overproduction near optimal yield (green curves). Genes where the blue and green distributions do not overlap are targets for modulation of gene expression. (TIFF) [file pcbi.1005140.s002.tiff]

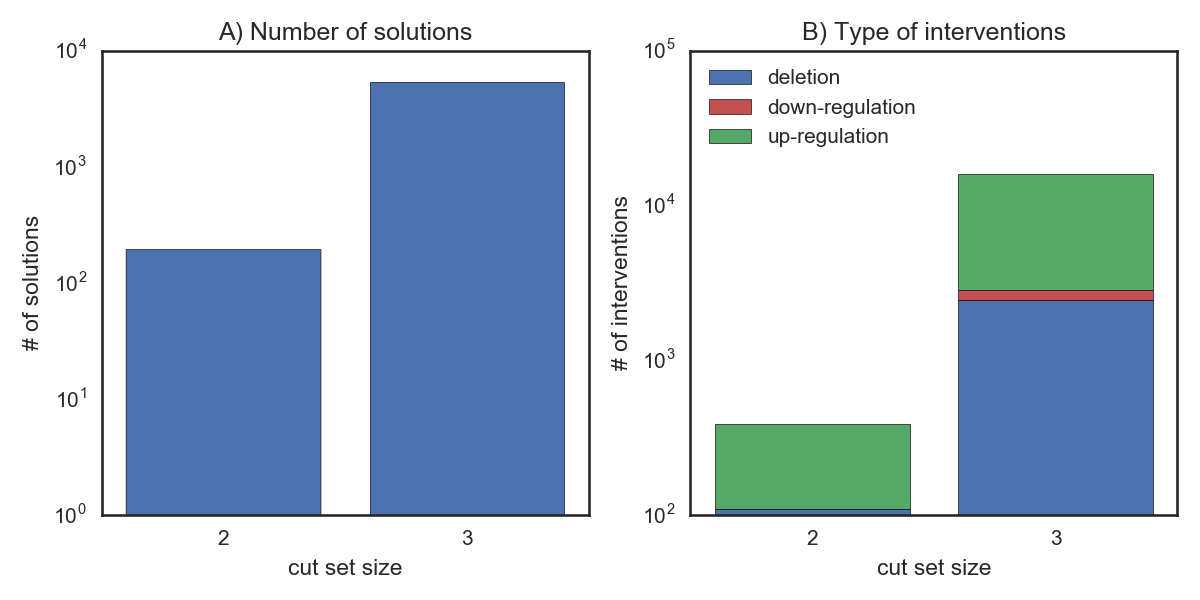

Supplement: S3 Fig — Gene-based strain design using cRegMCS for growth-coupled ethanol production in E. coli under anaerobic conditions (μ ≥ 0.001 h−1, Yeth/glc ≥ 1.4): a) size of the solution pool for each cut size; b) total number of interventions of each type in the solution pool. (TIFF) [file pcbi.1005140.s003.tiff]

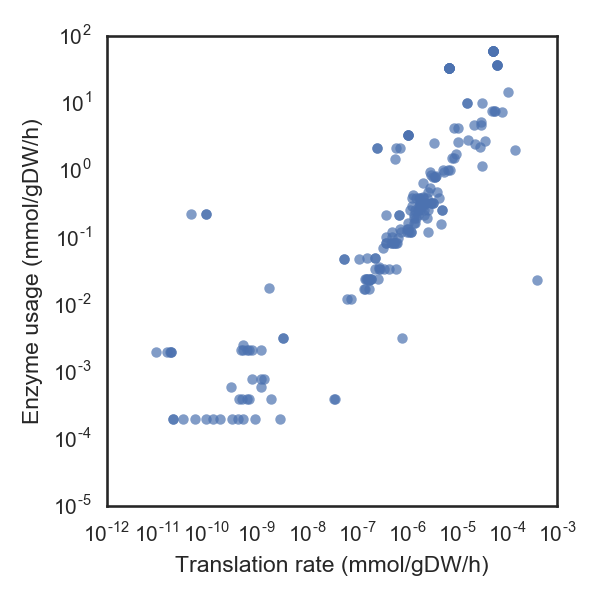

Supplement: S4 Fig — Comparison between the protein translation rates predicted by the ME-model and the respective enzyme usage predicted with gene-pFBA for a wild-type strain growing under aerobic conditions on glucose minimal medium with a glucose uptake rate of 10 mmol/gDW/h. (TIFF) [file pcbi.1005140.s004.tiff]
